# Timing of surgery after recovery from coronavirus disease 2019 (COVID-19) infection

**DOI:** 10.1017/ice.2020.325

**Published:** 2020-07-03

**Authors:** Rama Thyagarajan, Kristin Mondy

**Affiliations:** Department of Medicine, Division of Infectious Diseases, University of Texas at Austin, Dell Medical School, Austin, Texas

*To the Editor—*The coronavirus disease 2019 (COVID-19) pandemic has created unprecedented challenges for infection prevention experts, healthcare providers, and hospital administrative personnel. National, state, and local hospital leaders have had to rapidly adapt and update policies based on personal protective equipment and testing constraints. More recently, emerging data on presymptomatic transmission and prolonged viral shedding have created additional challenges.^1‐3^ As the pandemic continues and states begin to allow easing of nonemergent surgery restrictions, stakeholders are now faced with the task of developing policies for surgeries on recovered COVID-19–positive patients. Based on current data for COVID-19 and prior research on viral respiratory illnesses, we propose a rational surgical policy for such patients.

Most concerns with regard to surgery in recovered COVID-19 positive patients are related to the uncertainty of the postsymptomatic infectious period. Several studies have demonstrated prolonged or intermittent viral shedding in otherwise recovered, asymptomatic patients.^1‐3^

Accordingly, the CDC recently extended the time that recovered COVID-19 patients should remain isolated. Specifically, recovered patients should isolate for at least 10 days rather than 7 days after symptom onset and for at least 3 days after recovery.^4^ In its update, the CDC noted that replication-competent virus has not been successfully cultured >9 days after onset of illness, but data remains either unpublished or consists of very small cohorts.^3,4^ To this point, the CDC has recognized such uncertainty and has allowed consideration of a more stringent test-based strategy, citing “… circumstances under which there is an especially low tolerance for postrecovery SARS-CoV-2 shedding and risk of transmitting infection. In such circumstances, employers and local public health authorities may choose to apply more stringent recommendations, such as a test-based strategy, if feasible, or a requirement for a longer period of isolation after illness resolution.”^5^

Another important concern is risk of postoperative complications in newly recovered COVID-19 patients. In one study, cardiac surgery during influenza season was an independent risk factor for development of ARDS; duration of mechanical ventilation was also significantly longer than in noninfluenza seasons.^6^ Another large prospective study demonstrated that acute respiratory infection in the month preceding surgery was an independent risk factor for postoperative pulmonary complications.^7^ Finally, airway hyperreactivity following an upper respiratory infection may last 2–6 weeks and can contribute to surgical complications.^8^

A few case reports on outcomes have emerged on patients undergoing elective surgeries with confirmed active COVID-19 infections.^9,10^ A recent international multicenter cohort study of 1,128 COVID positive patients undergoing emergent (74%) and elective (26.1%) surgeries noted that pulmonary complications occurred in 51.2% of patients with a 30-day mortality of 38% (82% of all deaths were due to COVID-19 infection).^9^ The authors noted that during the COVID-19 outbreak in Wuhan, China, 34 patients who were likely asymptomatic with COVID-19 and undergoing elective procedures developed serious illness postoperatively. Postoperative ICU admissions were 44.1% with 20.5% COVID-related mortality within 4 weeks.^10^ The mortality rate was much higher than expected for similar surgeries in patients without COVID-19. It is unclear whether COVID-19 infection increased the odds of symptomatic severe viral infection and death by weakening the immune system or by triggering a hyperactive systemic immune response. The aforementioned publications and concern regarding healthcare personnel risk of acquiring COVID-19 in the surgery setting has led to routine preoperative COVID-19 testing and the delay of nonemergent surgery in many healthcare institutions.

These data raise important questions on timing of nonemergent surgery after asymptomatic and symptomatic COVID-19 infections. With wide availability of COVID-19 RT-PCR testing, many institutions are considering the use of a CDC test-based strategy to remove COVID-19 patients from isolation and clear them for surgery. Complicating this issue is the frequent persistence of positive PCR test results in patients for several weeks. Although infectivity is thought to be low, uncertainty remains, especially in the setting of aerosolizing procedures such as intubation.

The options to clear a recovered COVID-19 patient for surgery could be a symptom or test-based strategy. We recommend a hybrid strategy until more information is available. Surgical procedures should be delayed if possible for 4–6 weeks. We recommend against routine testing in this group as patients are likely not infectious anymore and risk of airway reactivity or ARDS will be reduced.^6,8^ Between 2 and 4 weeks after symptom onset, we recommend a test-based strategy due to insufficient data that recovered COVID patients are completely uninfectious.^1,4,5^ Data from China suggest that patients with severe symptoms have higher viral burden and prolonged viral shedding.^1^ For surgery completion <2 weeks after COVID-19 symptoms or diagnosis, we recommend no testing and operating under COVID isolation precautions in the operating room. Immunocompromised patients are likely to shed longer and may be at higher risk of other infectious or pulmonary complications.^5^ We suggest individualized case-based decisions by involved providers until more information is available.

In summary, a hybrid strategy for recovered COVID surgical patients will reinforce the culture of safety in the hospital environment and will reduce excess testing. Our recommendations will be limited in hospital settings where tests are not readily available, test turn-around is slow, or PPE constraints exist. Future studies are needed to examine the duration of infectivity to operating room staff by surgical patients affected by COVID-19, especially when intubation or other aerosolizing procedures are required. As more COVID-19–positive patients recover, it will also be important to perform postoperative studies related to timing of surgery and risk factors for poor outcomes.
